# Editorial: Network bioscience Volume II

**DOI:** 10.3389/fgene.2023.1256025

**Published:** 2023-07-26

**Authors:** Marco Antoniotti, Bud Mishra, Marco Pellegrini

**Affiliations:** ^1^ Data and Computational Biology Laboratory, Dipartimento di Informatica, Sistemistica e Comunicazione, Università Degli Studi di Milano-Bicocca, Milan, Italy; ^2^ B4-Bicocca Bioinformatics Biostatistics and Bioimaging Centre, Università Degli Studi di Milano-Bicocca, Milan, Italy; ^3^ Courant Institute of Mathematical Sciences, New York University, New York, NY, United States; ^4^ Consiglio Nazionale Delle Ricerche, Pisa, Italy

**Keywords:** network bioscience, gene regulatory networks, causal networks, knowledge graphs, metabolic graph models

Network biology is based on the intuition that the quantitative modeling and algorithmic tools of network theory offer new possibilities to understand, model, and simulate the cell’s internal organization and evolution, fundamentally altering our view of cell biology. As network biology has been gaining ground and recognition in the last 20 years, the scope of its application, while still well grounded in molecular biology and genetics, has moved steadily from tackling fundamental biological questions towards translational medicine, including modeling of diseases and applications in drug design and drug action prediction.

This Research Topic *Network Bioscience Vol II* follows in the track of the first one *Network Bioscience* completed in 2019 (Antoniotti et al., 2019), and it aims at collecting cutting-edge research on the many guises of network bioscience.

The papers contained in the present Research Topic are examples of how network and graph analysis can be used to elucidate various aspects of biological systems from inferring missing annotations, handling heterogeneous data types, including the vast literature available online, understanding metabolic dynamics, phenotype-genotype linking, to relationships assessment among diverse omics data for drug design and drug repositioning, to a deeper understanding of modularity in gene networks.

Among the recent trends with a potential of high impact, a most notable one is the incorporation of causality considerations and concepts within the classical network models so to make better use of perturbation data that are currently not exploited to their full potential. In particular such hybrid causal network models help bridging the gap between *descriptive* and *actionable network models*, the former successfully describe biological systems as they are, the latter allows us to formulate questions and find answers within the vast scope of *what-if*, counterfactual, worlds.

## Papers presentation

The papers collected in this Research Topic are roughly grouped as follows:

• “Foundational” papers,
• Analysis of particular biomedical problems,
• Algorithms and Tools.

Five foundational papers in this collection tackle in innovative ways basic issues in the mathematical modeling of bio-networks, covering an overview of relevant modularity concepts, to incorporating causality and biologically plausible sparsity assumptions in gene regulatory networks (GRNs), as well as empirically-found motif sub-network distributions.

Alcalá-Corona et al. bridge a notable gap between the perspective on community detection and network modularity derived from statistical physics and network science on the one hand, and its adaptation and application in biological research, on the other hand.

Maheshwari et al. present a general biological network inference method that combines the discovery of a parsimonious network structure and the identification of Boolean functions that determine the dynamics of the system. The method uses a causal logic framework to assimilate indirect information obtained from perturbation experiments and infer relationships that have not yet been documented experimentally.

Seçilmiş et al. note that many gene regulatory networks (GRNs) use sparsity definitions that are independent of other relevant biological properties expected from GRNs. They thus provide a general approach for identifying GRN that are both biologically accurate and structurally sparse GRN, within the entire space of possible GRNs, by selection criteria based on Akaike and Bayesian Information Criterion (AIC and BIC) adapted to the task of GRN inference.

Zhivkoplias et al. developed a novel motif-based preferential attachment algorithm, *FFLatt*, that aims at constructing a gene-proteins gene-regulatory network (GRN) rich in feed-forward loop (FFL) which are network motifs known to be significantly enriched in experimentally validated GRN.

In cancer driver gene identification, it is often assumed that a driver mutation is less likely to occur in case of an earlier mutation that has common functionality in the same molecular pathway (mutual exclusivity—ME). Ahmed et al. note that the current mutual exclusivity tests lack a network-centric view and thus fail to model key aspects of the problem. Thus they propose a network-centric framework to evaluate the pairwise significance values found by statistical ME tests and correct potential biases.

Two articles apply network techniques in the area of optimization of antibody design for drug design and to challenging modeling of the immune system’s role for a specific relevant complex condition (human infertility).

In the context of SARS-CoV-2 studies, Gross and Sharan tackle a fundamental problem of growing importance for antibody design that is the identification of mutations of concern of the Spike protein with high escape probability.

Taraschi et al. use a network-based approach to explore the etiopathogenic mechanisms involved in human hypofertility and infertility, aiming at understanding the involvement of the immune system.

Several of the articles describe advances in designing and providing the scientific community with increasingly powerful tools and algorithms capable of making the best use of the vast amount of heterogeneous and often noisy and incomplete biological data available from online repositories.

Castresana-Aguirre et al. note that when analyzing the association between a gene set and a pathway an issue that is generally ignored is that gene sets often represent multiple pathways. They experimentally found that pre-clustering of genes can be beneficial in this association studies by increasing the sensitivity of pathway analysis methods and by providing deeper insights into biological mechanisms related to the phenotype under study.

Di Maria et al. introduce *BioTAGME*, a system for the inference of novel knowledge and new hypotheses from the current biomedical literature analysis by constructing an extensive Knowledge Graph modeling relations among biological terms and phrases extracted from titles and abstracts of papers available in PubMed.

Semantic knowledge graphs (KGs) are increasingly used to combine unstructured human-curated full-text literature data and structured gene expression data from biomedical databases. In this area, Gurbuz et al. here demonstrate how KGs can be used to find new indications for existing drug targets in order to accelerate the process of launching a new drug for a disease on the market.

Often newly sequenced prokaryotic genomes have poor initial gene functional annotation and missing metabolism pathway gene assignments. To counter these shortcomings, Lu et al. developed *PPA-GCN*, a prokaryotic pathways assignment framework based on graph convolutional network, to assist functional pathway assignments.

Galvão Ferrarini et al. describe a novel tool, *Totoro*, that aims at predicting the metabolic reactions that are most likely active during the transient states of a metabolic network as a result of network perturbation simulations.

Zhao et al. propose a computational method called *LncPNet* to predict potential lncRNA–protein interactions based on spatial embedding a lncRNA–protein heterogeneous network into a collection of low-dimensional latent representations.
